# Robot-assisted navigation system for CT-guided percutaneous lung tumour procedures: our initial experience in Hong Kong

**DOI:** 10.1186/1470-7330-14-S1-S5

**Published:** 2014-10-09

**Authors:** CM Chu, SCH Yu

**Affiliations:** 1 Department of Imaging and Interventional Radiology The Chinese University of Hong Kong, Prince of Wales Hospital, Shatin, N.T., Hong Kong

## Purpose

To evaluate the new robot-assisted navigation system for CT-guided lung tumour procedures

## Materials and methods

Imaging-guided lung procedures are usually challenging due to patient breathing. This is an ongoing prospective study with 50 patients targeted in a university-based hospital. This was an initial assessment of efficacy involving 10 patients with lung tumours who underwent CT-guided lung interventions utilizing the robot-assisted Navigation system (Maxio, Perfint Healthcare, USA). The targeted needle pathway was planned on Maxio Robotic system based on pre-procedural CT-scans. The primary endpoint was satisfactory instrument position for intended intervention. Lesion size and depth from skin were noted. Performance level was documented on a five-point scale (5-1: excellent-poor). Total radiation doses were recorded and compared against 20 patients with conventional CT-guidance and CT-fluoroscopy lung procedures (ratio 1:1).

## Results

There were 7 male and 3 female patients in the robotic group. Average age was 72.1 years (range 67-78). 8 patients underwent lung biopsy while the rest had thermal ablation or fiducial marker insertion. Average lesion size was 2.8cm (range 1.9-4.1cm). Average lesion depth was 6.2cm (range 3.7-8.6cm). All interventions met the primary endpoint of satisfactory instrument positioning. Average performance levels were 4.5. Average radiation dose (Dose Linear Product) was 480.4 (range 196.5-959.8) whereas conventional CT-guidance was 645.4 (range 285.1-1043.5) and CT-fluoroscopy was 460.1 (range 214.2-1157.0).

## Conclusions

Our initial experience demonstrated effectiveness of the robot-assisted navigation system for CT-guided lung tumour interventions with lower radiation dose compared with conventional CT-guided procedures. Radiation doses were similar to CT-fluoroscopy without radiation exposure to interventional radiologists. Targeting success rate for satisfactory intervention was 100%.

